# Awareness and agreement with neurofibromatosis care guidelines among U.S. neurofibromatosis specialists

**DOI:** 10.1186/s13023-022-02196-x

**Published:** 2022-02-10

**Authors:** Vanessa L. Merker, Pamela Knight, Heather B. Radtke, Kaleb Yohay, Nicole J. Ullrich, Scott R. Plotkin, Justin T. Jordan

**Affiliations:** 1 Department of Neurology and Cancer Center, Massachusetts General Hospital, 55 Fruit St, Yawkey 9E, Boston, MA 02144 USA; 2 Center for Healthcare Organization and Implementation Research (CHOIR), VA Bedford Healthcare System, Bedford, MA 01730 USA; 3 Children’s Tumor Foundation, New York, NY 10017 USA; 4 Division of Genetics, Medical College of Wisconsin, Milwaukee, WI 53226 USA; 5 Department of Neurology, NYU Langone Health, New York, NY 10017 USA; 6 Department of Neurology, Boston Children’s Hospital, Boston, MA 02115 USA

**Keywords:** Neurofibromatosis 1, Neurofibromatosis 2, Schwannomatosis, Practice guidelines, Rare diseases, Implementation science

## Abstract

**Introduction:**

The neurofibromatoses (NF) are a group of rare, genetic diseases sharing a predisposition to develop multiple benign nervous system tumors. Given the wide range of NF symptoms and medical specialties involved in NF care, we sought to evaluate the level of awareness of, and agreement with, published NF clinical guidelines among NF specialists in the United States.

**Methods:**

An anonymous, cross-sectional, online survey was distributed to U.S.-based NF clinicians. Respondents self-reported demographics, practice characteristics, awareness of seven NF guideline publications, and level of agreement with up to 40 individual recommendations using a 5-point Likert scale. We calculated the proportion of recommendations that each clinician rated “strongly agree”, and assessed for differences in guideline awareness and agreement by respondent characteristics.

**Results:**

Sixty-three clinicians (49% female; 80% academic practice) across > 8 medical specialties completed the survey. Awareness of each guideline publication ranged from 53%-79% of respondents; specialists had higher awareness of publications endorsed by their medical professional organization (p < 0.05). The proportion of respondents who “strongly agree” with individual recommendations ranged from 17%-83%; for 16 guidelines, less than 50% of respondents “strongly agree”. There were no significant differences in overall agreement with recommendations based on clinicians’ gender, race, specialty, years in practice, practice type (academic/private practice/other), practice location (urban/suburban/rural), or involvement in NF research (p > 0.05 for all).

**Conclusions:**

We identified wide variability in both awareness of, and agreement with, published NF care guidelines among NF experts. Future quality improvement efforts should focus on evidence-based, consensus-driven methods to update and disseminate guidelines across this multi-specialty group of providers. Patients and caregivers should also be consulted to proactively anticipate barriers to accessing and implementing guideline-driven care. These recommendations for improving guideline knowledge and adoption may also be useful for other rare diseases requiring multi-specialty care coordination.

**Supplementary Information:**

The online version contains supplementary material available at 10.1186/s13023-022-02196-x.

## Introduction

The neurofibromatoses (NF)—neurofibromatosis 1 (NF1), neurofibromatosis 2 (NF2) and schwannomatosis—are rare genetic disorders that predispose patients to develop multiple nerve sheath tumors and many other physical, neurocognitive, and psychosocial symptoms [1]. Like many other rare, genetic diseases, NF presents with a varied clinical spectrum requiring care from multiple medical specialties. For example, patients with NF1 may visit pediatricians for initial evaluations; neurologists or neuro-oncologists for brain tumors, learning disabilities, headaches, or focal neurological deficits; plastic surgeons or dermatologists for cutaneous neurofibromas; orthopedic surgeons for scoliosis or pseudoarthrosis; and various other specialists for their complex disease. The need for specialty care across a wide range of manifestations may lead to fragmented care, highlighting the critical importance of research on how to disseminate clinical best practices in NF and other rare diseases.

Clinical practice guideline publications provide clinicians with collated recommendations based on systematic evidence reviews, while expert review documents provide recommendations where evidence-based data is limited [2]. Currently there are seven peer-reviewed publications providing clinical care recommendations for NF, hereafter referred to collectively as ‘NF guidelines’ [3–9]. Prior work has shown the importance of clinicians’ knowledge and attitudes—including their familiarity and agreement with guidelines—in predicting implementation of clinical guidelines [10, 11]. However, to date, there is no evidence of how broadly NF guidelines have been disseminated across the various specialties caring for NF patients, nor is there data on the agreement with or use of these guidelines. Therefore, we sought to evaluate the awareness of, and agreement with, NF guidelines among NF clinicians as a first step to developing consensus on NF clinical best practices.

## Methods

### Participant recruitment

United States based clinicians who currently care for neurofibromatosis patients were eligible to participate in the survey. Potentially eligible participants were identified using registration lists for the 2016–2018 Children’s Tumor Foundation NF conferences, the largest NF-specific research conference in the U.S. Registration lists did not reliably differentiate conference attendees’ clinical credentials nor their country of residence, so all potentially eligible participants (n = 358) were contacted and screening questions at the start of the survey were used to filter out non-clinicians and international participants. The chair of the Children’s Tumor Foundation Clinical Care Advisory Board emailed survey invitations in September 2019, with a follow-up reminder two weeks later. Participation in the survey was voluntary and anonymous. The study was deemed exempt by the Mass General Brigham Institutional Review Board.

### Survey design

This cross-sectional survey was administered online using REDCap, a secure online data collection platform [12]. Respondents were asked to self-report demographic data and practice characteristics [i.e. gender, race, ethnicity, medical specialty, years in practice, practice type (academic practice/private practice/other), practice location (urban/suburban/rural), involvement in NF research, affiliation with the Children’s Tumor Foundation NF Clinic Network, and whether their practice currently included pediatric and adult NF1, NF2, and schwannomatosis patients.] The research team identified six relevant NF guideline publications [3–8] via literature review to include in the survey (as French guidelines from Bergqvist et al. (2020) were published after this survey was administered). All respondents were asked to identify whether they were previously aware of each publication [which were identified by first author, title, journal, publication year, PubMed ID and primary topic area (i.e. pediatric NF1)].

40 individual recommendations were extracted from five guideline publications [4–8] for assessment of agreement (as pediatric NF1 guidelines from Miller et al. (2019) were published after this section of the survey was finalized). Recommendations were largely extracted verbatim, with minor changes in wording to combine nearly identical recommendations from multiple publications or to improve grammar (Table 1). Respondents were asked to rate their agreement with each individual recommendation relevant to their practice population using a 5-point Likert scale (e.g., only clinicians whose practice included pediatric NF1 patients were asked to rate pediatric NF1 recommendation). Of note, clinicians were not asked to rate their adherence to recommendation in their own clinical practice, only their level of personal agreement with the text provided. Respondents could also optionally provide free-text comments about any recommendation.

**Table 1:** NF clinical care recommendations assessed in the current study

	Citation
*Neurofibromatosis 1 (NF1) recommendations*	
MRI is preferred over CT scanning to reduce ionizing radiation exposure in patients with NF1	4
Blood pressure should be recorded at least annually in patients with NF1 from early childhood through adulthood	3, 5, 7
Patients with NF1 should be educated about malignant peripheral nerve sheath tumor (MPNST) signs and symptoms at initial and follow up visits	3, 4, 5, 7
Development and progress at school should be recorded at each annual visit for pediatric patients with NF1	3, 5
For hypertensive patients with NF1 who are under 30 years of age, pregnant and/or have abdominal bruits, causes of renovascular hypertension should be evaluated	4, 5
Height and weight should be recorded for patients with NF1 at every visit until one year old, then at least annually until adulthood	3, 5, 7
Patients with NF1 under 8 years old should have annual testing of visual acuity and fundoscopy to assess for optic disc pallor and elevation	5
Neurologic examination should be performed routinely for patients with NF1 from one month to one year of age, then annually until adulthood	3, 5, 7
Pubertal development should be recorded for patients with NF1 at each annual visit from early childhood through puberty	3, 5, 7
Evaluation of the skin of patients with NF1 should be recorded at each visit until 1 year old, and then at least annually thereafter	3, 5, 7
From birth to 8 years old, patients should have ophthalmologic exams every 6–12 months including objective and quantitative visual acuity testing, visual fields, pupillary reflexes, and fundus exam	7
Head circumference should be recorded for pediatric patients with NF1 at each visit until puberty	3, 5
Patients with NF1 should be referred to orthopedics if there is concern about scoliosis	4, 5
Patients with NF1 should be seen at least annually at an NF clinic	3
Patients with NF1 should be followed at a specialized NF clinic	4
All individuals with NF1 should have annual clinical evaluation of the back with Adam's forward bend test	3, 4, 5
Adult patients with NF1 should be screened for depression	4
Women with NF1 should have annual mammogram starting at age 30 years, and consideration of contrast-enhanced breast MRI between ages 30 and 50 years	4, 7
Family planning should be revisited annually for patients of child bearing age who have NF1	4
Patients with NF1 should be supplemented with vitamin D to reach serum 25-hydroxyvitamin D concentrations in the sufficient range	4
For hypertensive patients with NF1 who are under 30 years of age, pregnant and/or have abdominal bruits, concomitant screening for pheochromocytoma with plasma free metanephrines is recommended	4
MRA is the preferred imaging modality for evaluation of renovascular hypertension. However, for patients with impaired renal function, spiral CT and CT angiography may be used	4
Pregnant women with NF1 should be referred to a high-risk obstetrician	4
Adults with NF1 should be asked about chronic fingertip and toe pain in the assessment of possible glomus tumors	4
Because the risk of MPNST being associated with high internal tumor burden, whole-body MRI should be considered between ages of 16 and 20 years to assess this in patients with NF1	7
Preanesthesia neuraxial imaging to evaluate for spinal or paraspinal neurofibromas is probably not needed. If there are concerns, spinal anesthesia may be considered	4
*Neurofibromatosis 2 (NF2) Recommendations*	
Patients with NF2 should be followed at a specialized NF clinic	6
Patients with NF2 should be seen at least annually at an NF clinic	8
Ophthalmologic examination by a specialized ophthalmologist is recommended in children with NF2	8
Annual audiology with measurement of pure-tone thresholds and word recognition scores is recommended for patients with NF2	8
All children presenting with either clear diagnostic criteria for NF2, or those with an NF2 tumor (any schwannoma or meningioma) presenting in childhood should undergo genetic testing of NF2	8
Patients with NF2 should be informed that follow-up for life with interval scanning is necessary	6
Annual brain MRI is recommended for patients with NF2, unless no tumors are seen on first scan in which case frequency may reduce to every 2 years	8
Surveillance spinal MRI is recommended for patients with NF2 at 24- to 36-month intervals, unless there are no tumors in which case the frequency can be decreased	8
Surveillance spinal MRI is recommended for patients with NF2 at 24- to 36-month intervals beginning at 10 years of age	8
The interval between spinal surveillance MRI scans may be increased in patients with NF2 if there is no disease detected on baseline imaging	8
Annual brain MRI is recommended for patients with NF2 starting at 10 years of age, unless no tumors are seen on first scan in which case frequency may reduce to every 2 years	8
*Schwannomatosis Recommendations*	
Test for pathogenic variants ('mutations') in *SMARCB1* and *LZTR1* genes should be performed in children and young adults with one or more non-intradermal schwannoma, including those with vestibular schwannoma negative for NF2	8
Baseline MRI of the brain should be obtained at diagnosis, then every 2–3 years, beginning at age 10 for *SMARCB1*-mutant patients and at age 15–19 for *LZTR1*-mutant patients with schwannomatosis	8
Baseline MRI of the spine should be obtained at diagnosis, then every 2–3 years, beginning at age 10 for *SMARCB1*-mutant patients and at age 15–19 for *LZTR1*-mutant patients with schwannomatosis	8

3 – Miller et al. (2019); 4 – Stewart et al. (2018); 5 – Ferner et al. (2007); 6 – Evans et al. (2005); 7 – Evans et al. (2017); 8 – Evans et al. (2017)

### Data analysis

Descriptive data on all variables are reported as frequencies and percentages. Only questions assessing study eligibility and determining the subpopulations of NF included in each clinicians’ practice were mandatory; all other questions were optional, and percentages are reported out of the sample size of non-missing responses unless otherwise noted. Due to the risk of acquiescence bias (the tendency for survey respondents to select positive response options) [13], we focused our reporting of agreement on the percentage of respondents who selected “strongly agree”, thus actively indicating a strong preference in favor of each recommendation. As clinicians’ level of support for a clinical practice guideline has been associated with their adherence to guidelines in practice [14, 15], this type of top-box analysis—used often in patient experience surveys [16–18]—may best predict clinicians’ real-world behavior. We calculated 95% confidence intervals for the percentages of respondents who strongly agreed with each recommendation; average margin of error across all recommendation was 12.7% (Supplementary Information).

We examined whether clinician demographics or practice characteristics were correlated with level of agreement with NF clinical recommendation. Given the large number of individual recommendations and respondent subgroups of interest (and concomitant risk of Type 1 error from performing multiple comparisons), we assessed clinicians’ overall agreement with all recommendations relevant to their practice population. To do this, we calculated the proportion of recommendations to which each clinician ‘strongly agreed’ out of the total number of recommendations for which they completed ratings. This proportion could range from 0 (does not strongly agree with any recommendation) to 1 (strongly agrees with all recommendations). This proportion was compared across respondent subgroups using t-tests or ANOVA as appropriate.

Two NF1 guidelines publications were endorsed by U.S. medical societies—Miller et al. guidelines for pediatric NF1 published by the American Academy of Pediatrics (AAP) and endorsed by the American College of Medical Genetics and Genomics [3] (ACMG) and Stewart et al. guidelines for adult NF1 published by ACMG [4]. To better understand guideline dissemination patterns, we assessed whether clinicians in these specialties were more likely to report being aware of guideline publications than clinicians in other specialties using Fisher’s exact test. A p-value of ≤ 0.05 was considered statistically significant for all tests. Finally, we used qualitative content analysis to summarize respondent’s free-text comments [19].

## Results

Eighty-two U.S. based clinicians accessed the survey; 63 completed at least one agreement rating and comprised our final analytic sample [63/358 (17.6%) of potentially eligible respondents, 63/82 (76.8%) of eligible respondents who accessed the survey]. Clinician demographics, practice characteristics, and NF specialization are shown in Table 2. Clinicians represented > 8 medical or surgical specialties, with one-third (n = 21) of respondents in neurology or neuro-oncology. Clinicians were located across 26 U.S. states with the greatest number of respondents from New York, California, Florida, Massachusetts, and Minnesota (respectively). Most respondents (n = 50, 79.4%) provided clinical care as part of a specialized neurofibromatosis clinic affiliated with the Children’s Tumor Foundation NF Clinic Network [20] and the majority were involved in NF research (n = 39 for clinical trials and n = 47 for other research studies).

**Table 2:** Respondent demographics and practice characteristics (n = 63)

	N (%)
*Clinician demographics*	
Gender	
Female	31 (49.2%)
Male	27 (42.9%)
Non-binary	1 (1.6%)
Prefer not to answer or missing	4 (6.3%)
Race	
White	53 (84.1%)
Black or African American	1 (1.6%)
Asian	6 (8.4%)
American Indian or Alaska Native	0 (0.0%)
Native Hawaiian or other Pacific Islander	0 (0.0%)
Other	2 (3.2%)
Missing	1 (1.6%)
Ethnicity	
Hispanic or Latino	3 (4.8%)
Not Hispanic or Latino	56 (88.9%)
Missing	4 (6.3%)
Primary specialty	
Medical genetics	18 (28.6%)
Neuro-oncology	11 (17.4%)
Neurology	10 (15.9%)
Pediatrics	5 (7.9%)
Hematology/oncology or medical oncology	5 (7.9%)
Dermatology	2 (3.2%)
Neurosurgery	1 (1.6%)
Orthopedic surgery	0 (0.0%)
Other	8 (13.7%)
Missing	3 (4.8%)
Years in post-training practice	
< 5 years	11 (17.4%)
5–10 years	14 (22.2%)
10–20 years	21 (33.3%)
> 20 years	17 (27.0%)
*Practice characteristics*	
Practice type	
Academic medical practice	50 (79.4%)
Private practice	5 (7.9%)
Other	7 (11.1%)
Missing	1 (1.6%)
Location of primary office	
Urban	49 (77.8%)
Suburban	12 (19.0%)
Rural	1 (1.6%)
Missing	1 (1.6%)
Primary language of patients in practice	
English	61 (96.8%)
Spanish	2 (3.2%)
*Neurofibromatosis specialization*	
Affiliation with children’s tumor foundation NF clinic network	
Yes	50 (79.4%)
No	11 (17.4%)
Unsure	1 (1.6%)
Missing	1 (1.6%)
Clinician involvement in NF treatment trials	
Yes	39 (61.9%)
No	23 (36.5%)
Missing	1 (1.6%)
Clinician involvement in NF non-treatment research	
Yes	47 (74.6%)
No	16 (25.4%)
Clinicians seeing each patient population	
Pediatric neurofibromatosis 1	55 (87.3%)
Adult neurofibromatosis 1	51 (81.0%)
Pediatric neurofibromatosis 2	48 (76.2%)
Adult neurofibromatosis 2	36 (57.1%)
Schwannomatosis	36 (57.1%)

### Guideline awareness

Clinicians self-reported their awareness of NF guideline publications (Table 3). The percentage of respondents who were aware of each guideline set ranged from 53.2% (for schwannomatosis guidelines within Evans et al. 2017) to 79.4% (for NF1 guidelines published by Ferner et al. 2007). Among those respondents who reported currently seeing the relevant patient population, awareness was only marginally increased (by 1.1 to 5.1 percentage points), with the exception of adult NF2 guidelines by Evans et al. 2005, in which awareness increased 11.6 percentage points to 65.7% of respondents. Medical geneticists were significantly more likely to report awareness of adult NF1 guidelines endorsed by the ACMG than clinicians of other specialties (100% of medical geneticists vs. 56.1% of other specialists, p = 0.0008). Pediatricians and medical geneticists were also more likely to report awareness of pediatric NF1 guidelines endorsed by the AAP and the ACMG (87.0% of pediatricians and medical geneticists vs. 55.6% of other specialists, p = 0.021).

**Table 3:** Clinicians’ self-reported awareness of NF clinical guideline publications

	Total number of respondents (N)	Aware of guideline document N (%)	Unaware of guideline document N (%)	Unsure N (%)
*Neurofibromatosis 1 Guidelines*				
Stewart et al. (2018)	62	43 (69.4%)	16 (25.8%)	3 (4.8%)
Ferner et al. (2007)	63	50 (79.4%)	11 (17.5%)	2 (3.2%)
Evans et al. (2017a)	63	47 (58.7%)	21 (33.3%)	5 (7.9%)
Miller et al. (2019)	62	43 (69.4%)	17 (27.4%)	2 (3.2%)
*Neurofibromatosis 2 Guidelines*^a^				
Evans et al. (2005)	61	33 (54.1%)	26 (42.6%)	2 (3.3%)
Evans et al. (2017b)	61	40 (65.6%)	18 (29.5%)	3 (4.9%)
*Schwannomatosis Guidelines*^a^				
Evans et al. (2017b)	62	33 (53.2%)	22 (35.5%)	7 (11.3%)

^a^Respondents were asked to rate their awareness of the neurofibromatosis 2 guidelines and schwannomatosis guideline within Evans et al. 2017b separately

### Agreement with recommendations for NF clinical care

Overall, at least half of survey respondents strongly agreed with 24/40 (60%) of NF recommendations. The median proportion of recommendations with which each clinician strongly agreed was 0.55 (range 0–1; 25th–75th percentile, 0.35 to 0.73). There were no statistically significant differences in recommendation agreement proportion based on clinicians’ demographic or practice characteristics.

**Fig. 1:**
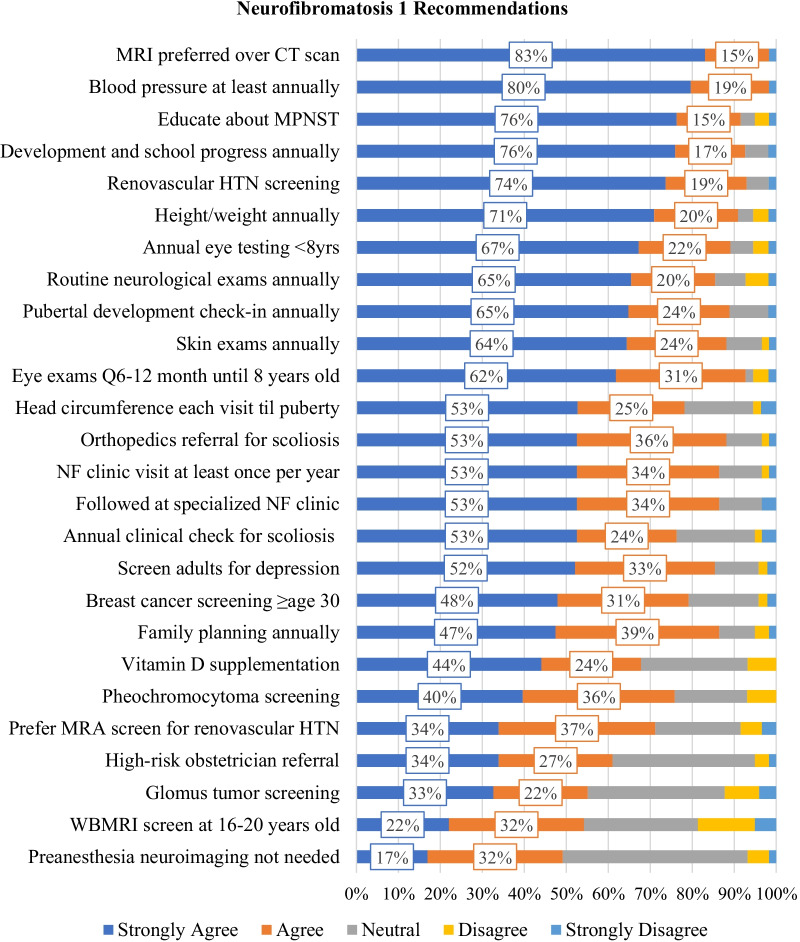
NF Clinician Agreement with Neurofibromatosis 1 Recommendations. Stacked bar charts displaying the percentage of clinicians who “strongly agreed” (dark blue), “agreed” (orange), were “neutral” (gray), “disagreed” (yellow), or “strongly disagreed” (light blue) with each recommendation. Overlaid boxes display the percentage of clinicians who “strongly agreed” (dark blue) and “agreed” (orange) with each recommendation, rounded to the nearest percentage point. Recommendations are presented with short descriptions for reference; for full text and citations please refer to Table 1, where all recommendations are presented in the same rank order. CT = computerized tomography; HTN = hypertension; NF = neurofibromatosis; MPNST = malignant peripheral nerve sheath tumor; MRA = magnetic resonance angiography; MRI = magnetic resonance imaging; WBMRI = whole body magnetic resonance imaging

Clinicians’ level of agreement with NF1 recommendations are presented in Fig. 1 and Additional file 1: Table S1. Level of strong agreement with individual NF1 recommendations ranged from 17 to 83%. Strong agreement was highest for the preference of MRIs over CT scans to reduce exposure to ionizing radiation exposure (83%); annual blood pressure checks (80%); education about signs and symptoms of malignant peripheral nerve sheath tumors (76%); and annual check-ins on development and school progress for pediatric patients (76%). For 9/26 (34.6%) of NF1 recommendations, less than half of respondents selected ‘strongly agree’. These recommendations addressed breast cancer screening, counseling regarding family planning, vitamin D supplementation, evaluation of hypertension, pregnancy management, assessment of glomus tumors, and use of whole-body MRI. Of note, only one of these nine recommendations was endorsed by more than one guideline publication, while essentially all of the highest ranked recommendations were endorsed by multiple guideline publications.

**Fig. 2:**
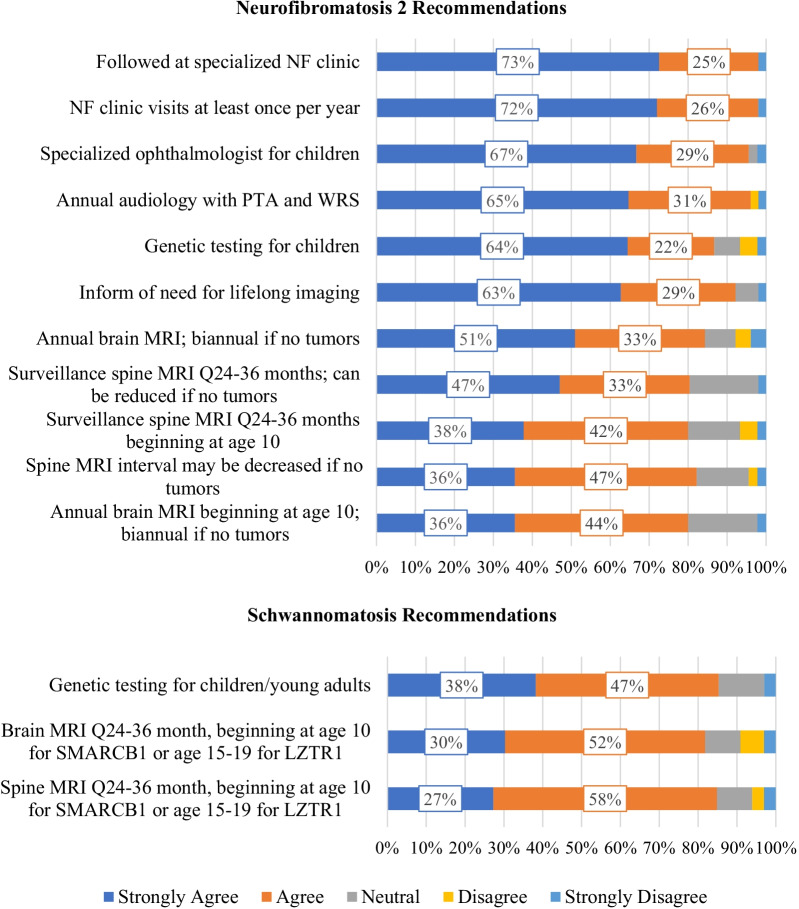
NF Clinician Agreement with Neurofibromatosis 2 and Schwannomatosis Recommendations. Stacked bar charts displaying the percentage of clinicians who “strongly agreed” (dark blue), “agreed” (orange), were “neutral” (gray), “disagreed” (yellow), or “strongly disagreed” (light blue) with each recommendation. Overlaid boxes display the percentage of clinicians who “strongly agreed” (dark blue) and “agreed” (orange) with each recommendation, rounded to the nearest percentage point. Recommendations are presented with short descriptions for reference; for fulltext and citations please refer to Table 1, where all recommendations are presented in the same rank order. NF = neurofibromatosis; MRI = magnetic resonance imaging; PTA = pure tone average; WRS = word recognition score. *SMARCB1* and *LZTR1* refer to gene variants known to cause schwannomatosis

Clinicians’ level of agreement with NF2 and schwannomatosis recommendations are presented in Fig. 2 and Additional file 1: Table S1. Level of strong agreement with individual NF2 recommendations ranged from 36 to 73%. Strong agreement was highest for receiving care at specialized NF clinics (73%) and for receiving care annually at an NF clinic (72%). For 4/11 (36.4%) of NF2 recommendations, less than half or respondents selected ‘strongly agree’. These recommendations addressed the frequency of spinal MRIs in all patients and the timing of brain MRIs for pediatric patients. Agreement was noticeably lower for pediatric imaging recommendations including the provision to start surveillance at age 10 when compared to identical adult guidelines (absolute difference of 15.4 percentage points for brain MRIs and 9.3 percentage points for spinal MRIs). Level of strong agreement with three schwannomatosis recommendations addressing genetic testing for younger patients with potential schwannomatosis and the timing of brain and spine MRIs ranged from 27 to 38%.

Twenty optional free-text comments regarding NF recommendations were collected from eleven survey respondents. While there were insufficient comments to robustly explain disagreement with individual recommendations, categories of comments may illuminate potential sources of disagreement with NF guidelines in general. Comments were categorized into five main groups, in descending order of frequency: (1) disagreement about the timing of services recommended by a guideline; (2) disagreement that a guideline should apply to the entire population equally; (3) perception that guidelines were not in the provider’s scope of practice; (4) confusion about the way a guideline was worded; and (5) suggested addition to a guideline.

Some participants advocated for more frequent monitoring (e.g. “I think the eye exam with [visual fields/visual acuity] should be every 6 months until age 6, and then yearly”) or less frequent monitoring (e.g., “I think monthly [neuro] evals would be too frequent. If a baby seems to be doing well at the initial visit, then I see them at 6 months. If I have concerns about development, then I see them at 3 months.”) Similarly, multiple commenters argued that lower risk individuals need less intensive monitoring or fewer referrals (e.g., “I don't always refer to Ortho for scoliosis if it is mild and not progressing”) while higher-risk individuals may need more intensive monitoring (e.g. “If the patients family has an history of aggressive NF2, [starting] imaging earlier than 10 years is recommended.”) Some comments reflected that not all NF providers treat every NF complication or see a sufficient number of cases to feel prepared to assess the appropriateness of certain recommendations (e.g. “I am not up to date with NF2 and schwannomatosis, as I do not see adults nor many pediatric patients with these disorders, so I answer "neutral" in those cases”), and that some recommendations seemed more appropriate for non-specialists to oversee (e.g. “Some screening such as mammograms are part of routine healthcare.”) Finally, some comments reflected confusion about the way recommendations were worded (e.g., whether NF1 preanesthesia imaging guidelines were restricted to pregnant individuals).

## Discussion

We evaluated NF specialists’ awareness of and agreement with NF clinical guidelines to assess the efficacy of guideline dissemination to date and identify any areas of disagreement regarding best practices for NF clinical care. We identified wide variability in both awareness of, and agreement with, published NF guidelines. While many respondents were aware of relevant publications, and most recommendations did not have large proportions of disagreement, our findings highlight areas warranting further attention as the multidisciplinary NF community strives to reach consensus on optimal care practices. Approximately one-quarter to one-third of respondents were unaware of recently published NF guidelines, and the majority of survey respondents did not strongly agree with 40% of assessed recommendations for care. Recommendations applicable only to adults with NF1 (rather than recommendations applicable to all ages or only children with NF1); recommendations for imaging guidelines in NF2; and all schwannomatosis recommendations tended to have the lowest proportion of respondents who selected “strongly agree”. While clinician’s primary specialty was related to awareness of NF guidelines, no demographic or practice characteristics were associated with clinicians’ overall level of agreement with NF recommendations.

Clinicians’ familiarity with clinical guidelines is the first step in providing guideline-driven care, but can be inhibited by the large volume of information each clinician must process and the limited time they have available to stay informed with literature [10]. Our findings suggest that clinicians may be more aware of guidelines endorsed by their affiliated medical specialty organization. Given the multi-system manifestations of NF and diverse array of specialists involved in NF care, the field should evaluate strategies for broader dissemination of existing guidelines across specialties. Prior research suggests a multifaceted dissemination approach using both written materials (e.g. journal articles, checklists, educational materials, etc.) and personal appeals (e.g. via local forums or one-on-one contacts from peers) may be most effective [21, 22]. Rather than duplicate prior guideline creation efforts, medical societies relevant to NF may also want to review, endorse, and republish relevant guidelines to facilitate multidisciplinary collaboration in NF patient care.

However, there remains multiple NF care recommendations lacking strong agreement. Potential sources of this reduced enthusiasm are several-fold, as highlighted by qualitative comments and published literature, and other rare disease communities seeking to develop clinical guidelines should be aware of these pitfalls so as to proactively avoid them if possible. Perceived lack of evidence on efficacy or utility of a guideline-recommended assessment may limit agreement. For example, the utility of whole-body MRI is under study and routine use is increasing; however, the optimal use, timing and interpretation of this technology is not yet known [4]. Furthermore, not all medical centers have this imaging capability nor do all insurance plans cover this service. An inability to reliably implement a guideline-recommended assessment may also lead to lower levels of endorsement from clinicians. There may also be lower agreement where standardized guidelines conflict with individualized patient preferences (such as when personalized shared-decision making is recommended to determine optimal breast cancer screening schedules in women with NF) [4]. Finally, agreement ratings may be impacted by lack of clarity as to which population or clinical context is intended.

Our empirical data on agreement with NF clinical guidelines among U.S. specialists can inform discussions on the value of, evidence base for, and implementation of these guidelines. For recommendations with weaker agreement, consensus-based methods should be used to review guidelines, understand opposition to recommended health services, and propose any necessary clarifications and revisions.

Any efforts to update guidelines in NF or other rare disease should adhere to best practices for guideline development [23]. For example, the Appraisal of Guidelines, Research and Evaluation (AGREE II) instrument can be used to assess the quality of existing clinical guidelines and provide guidance on appropriate development and reporting of revised guidelines [24]. Many sources emphasize writing clear, unambiguous guidelines that are explicitly and transparently linked to the underlying evidence base [25, 26]. For recommendations with insufficient evidence to support widespread adoption, funders could prioritize systematic evidence reviews and/or additional prospective studies on the value of recommended interventions.

Recent research also emphasizes the need to incorporate patient perspectives in guideline development [23, 24]. This will be important to address patient preferences that may conflict with clinician recommendations and to address real-world barriers to guideline implementation such as limited access to specialists, lack of time/resources within clinic appointments, or issues with insurance coverage [27]. Input from clinicians, patients, and family members from diverse practice settings and geographic locations should thus be sought to pre-emptively understand access to and barriers to receiving guideline-driven care. A survey of patients and their caregiver to assess receipt of guideline-concordant care was recently launched within an NF patient registry for this purpose [28]. Furthermore, efforts to disseminate information about clinical guidelines directly to patients may prove fruitful in increasing guideline-concordant care, as shown in other rare diseases such as Down Syndrome [29].

## Limitations

As we desired broad input from the NF community on current guidelines, our recruitment email was sent to a large distribution list and the exact number of eligible clinicians approached and their response rate is unknown. It is possible that clinicians who see fewer NF patients or those with less knowledge of NF guidelines were less likely to respond to the survey. As such, the percentage of U.S. NF clinicians who agree with specific recommendations likely varies within our sample’s reported margin of error. Nonetheless, our findings highlight important areas of significant disagreement that need to be addressed. Our target population was clinicians attending NF-specific research conferences, who likely have a strong interest in NF and an established caseload of NF patients. This population is likely more aware of NF guideline publications and possibly more likely to agree with guidelines than primary care clinicians or non-NF specialists. Our sample also did not equally represent all medical specialties involved in NF care, nor did we distinguish whether clinicians had a high or low absolute volume of NF patients. Future work should examine how different clinician groups find and use guidelines for patients with rare diseases, and how specialists and primary care clinicians can share responsibilities for providing guideline-concordant care for such patients (especially for routine monitoring and surveillance). Finally, due to the large number of clinical care recommendations relative to our sample size, we did not statistically test for demographic or practice factors that may influence agreement with individual recommendations. Qualitative work exploring clinicians’ reasons for not strongly agreeing with specific recommendations and quantitative work exploring the degree to which clinicians adhere to guidelines in practice would be informative in revising guidelines and developing additional interventions to promote guideline use.

## Conclusions

In conclusion, our study demonstrates that there is significant variation in levels of strong agreement with NF clinical guidelines. For recommendations with strong agreement, future efforts to enhance guideline use should prioritize broad dissemination of guidelines across medical specialties. A focus on implementation of guidelines in routine care—for example, by providing multiple versions of guidelines adapted for different users and purposes, and organizing and formatting content to make it easy to understand and implement—will enhance the success of dissemination efforts [30]. For recommendations with weak or mixed agreement, future efforts to improve guideline use should prioritize evidence-based, consensus-driven approaches to reviewing and updating guidelines. An international Delphi panel, such as that used recently to revise NF diagnostic criteria, may be an efficient approach to gather widespread input from all relevant stakeholders, including patients and their family members [31]. Together these initiatives could help combat common barriers to guidelines awareness, agreement, and implementation, maximizing the chances for guideline adherence and the delivery of high-quality NF care in the future.

## Supplementary Information

**Additional file 1**. Clinicians’ level of agreement with individual published NF1, NF2, and schwannomatosis care recommendations.

## Data Availability

The datasets generated and analysed during the current study are not publicly available due to risk of identification from unique combinations of multiple demographic factors in this small group of specialists but de-identified data is available from the corresponding author on reasonable request.

## References

[1] Plotkin SR, Wick A (2018). Neurofibromatosis and schwannomatosis. Semin Neurol.

[2] Institute of Medicine (US) Committee on Standards for Developing Trustworthy Clinical Practice Guidelines, Graham R, Mancher M, Miller Wolman D, Greenfield S, Steinberg E. Clinical Practice Guidelines We Can Trust. Washington DC: National Academies Press; 2011. 24983061

[3] Miller DT, Freedenberg D, Schorry E (2019). Health supervision for children with neurofibromatosis type 1. Pediatrics.

[4] Stewart DR, Korf BR, Nathanson KL, Stevenson DA, Yohay K (2018). Care of adults with neurofibromatosis type 1: a clinical practice resource of the American College of Medical Genetics and Genomics (ACMG). Genet Med.

[5] Ferner RE, Huson SM, Thomas N (2007). Guidelines for the diagnosis and management of individuals with neurofibromatosis 1. J Med Genet.

[6] Evans DG, Baser ME, O'Reilly B (2005). Management of the patient and family with neurofibromatosis 2: a consensus conference statement. Br J Neurosurg.

[7] Evans DGR, Salvador H, Chang VY (2017). Cancer and central nervous system tumor surveillance in pediatric neurofibromatosis 1. Clin Cancer Res.

[8] Evans DGR, Salvador H, Chang VY (2017). Cancer and central nervous system tumor surveillance in pediatric neurofibromatosis 2 and related disorders. Clin Cancer Res.

[9] Bergqvist C, Servy A, Valeyrie-Allanore L, Ferkal S, Combemale P, Wolkenstein P (2020). Neurofibromatosis 1 French national guidelines based on an extensive literature review since 1966. Orphanet J Rare Dis.

[10] Cabana MD, Rand CS, Powe NR (1999). Why don't physicians follow clinical practice guidelines? A framework for improvement. JAMA.

[11] Fischer F, Lange K, Klose K, Greiner W, Kraemer A. Barriers and strategies in guideline implementation—a scoping review. 2016;4(3):36. 10.3390/healthcare4030036 PMC5041037 27417624

[12] Harris PA, Taylor R, Thielke R, Payne J, Gonzalez N, Conde JG (2009). Research electronic data capture (REDCap)—a metadata-driven methodology and workflow process for providing translational research informatics support. J Biomed Inform.

[13] Krosnick JA (1991). Response strategies for coping with the cognitive demands of attitude measures in surveys. Appl Cogn Psychol.

[14] Francke AL, Smit MC, de Veer AJE, Mistiaen P (2008). Factors influencing the implementation of clinical guidelines for health care professionals: a systematic meta-review. BMC Med Inform Decis Mak.

[15] Bierbaum M, Rapport F, Arnolda G (2020). Clinicians’ attitudes and perceived barriers and facilitators to cancer treatment clinical practice guideline adherence: a systematic review of qualitative and quantitative literature. Implement Sci.

[16] Lapin BR, Honomichl RD, Thompson NR (2019). Association between patient experience with patient-reported outcome measurements and overall satisfaction with care in neurology. Value Health.

[17] Hanson KT, Zalewski NL, Hocker SE, Caselli RJ, Habermann EB, Thiels CA (2018). At the intersection of patient experience data, outcomes research, and practice: analysis of HCAHPS scores in neurology patients. Mayo Clin Proc Innov Qual Outcomes.

[18] Indovina K, Keniston A, Reid M (2016). Real-time patient experience surveys of hospitalized medical patients. J Hosp Med.

[19] Hsieh HF, Shannon SE (2005). Three approaches to qualitative content analysis. Qual Health Res.

[20] Merker VL, Dai A, Radtke HB, Knight P, Jordan JT, Plotkin SR (2018). Increasing access to specialty care for rare diseases: a case study using a foundation sponsored clinic network for patients with neurofibromatosis 1, neurofibromatosis 2, and schwannomatosis. BMC Health Serv Res.

[21] Grol R. Successes and failures in the implementation of evidence-based guidelines for clinical practice. Med Care. 2001;39(8 Suppl 2):II46–54. 10.1097/00005650-200108002-00003. 10.1097/00005650-200108002-00003 11583121

[22] Grimshaw JM, Thomas RE, MacLennan G, et al. Effectiveness and efficiency of guideline dissemination and implementation strategies. Health Technol Assess. 2004;8(6):iii–iv, 1–72. 10.3310/hta8060. 10.3310/hta8060 14960256

[23] Montori VM, Brito JP, Murad MH (2013). The optimal practice of evidence-based medicine: incorporating patient preferences in practice guidelines. JAMA.

[24] Brouwers MC, Kho ME, Browman GP (2010). AGREE II: advancing guideline development, reporting and evaluation in health care. CMAJ.

[25] Lugtenberg M, Zegers-van Schaick JM, Westert GP, Burgers JS (2009). Why don't physicians adhere to guideline recommendations in practice? An analysis of barriers among Dutch general practitioners. Implement Sci.

[26] Pronovost PJ (2013). Enhancing physicians' use of clinical guidelines. JAMA.

[27] Kim C, Berta WB, Gagliardi A (2020). Exploring approaches to identify, incorporate and report patient preferences in clinical guidelines: Qualitative interviews with guideline developers. Patient Educ Couns.

[28] Seidlin M, Holzman R, Knight P (2017). Characterization and utilization of an international neurofibromatosis web-based, patient-entered registry: an observational study. PLoS ONE.

[29] Chung J, Donelan K, Macklin EA (2021). A randomized controlled trial of an online health tool about Down syndrome. Genet Med.

[30] Gagliardi AR, Brouwers MC, Palda VA, Lemieux-Charles L, Grimshaw JM (2011). How can we improve guideline use? A conceptual framework of implementability. Implement Sci.

[31] Legius E, Messiaen L, Wolkenstein P (2021). Revised diagnostic criteria for neurofibromatosis type 1 and Legius syndrome: an international consensus recommendation. Genet Med.

